# Characterization of extracellular matrix macromolecules in primary cultures of equine keratinocytes

**DOI:** 10.1186/1746-6148-6-16

**Published:** 2010-03-15

**Authors:** Michelle B Visser, Christopher C Pollitt

**Affiliations:** 1 The Australian Equine Laminitis Research Unit, School of Veterinary Science, University of Queensland, St Lucia, 4072, Australia

## Abstract

**Background:**

Most research to date involving laminins and extracellular matrix protein function in both normal and pathological conditions involves *in vitro *culture of keratinocytes. Few methods are established to allow for prolonged propagation of keratinocytes from equine tissues, including the hoof lamellae. In this study we modified cell isolation and culture techniques to allow for proliferation and sub-culturing of equine lamellar keratinocytes. Additionally, the production and processing of extracellular matrix molecules by skin and lamellar keratinocytes were studied.

**Results:**

Physical and proteolytic tissue separation in combination with media containing a calcium concentration of 0.6 mM in combination with additional media supplements proved optimal for proliferation and subculture of equine lamellar keratinocytes on collagen coated substratum. Immunofluorescence and immunoblotting studies confirmed that equine skin and lamellar keratinocytes produce Ln-332 *in vitro *and processing of this molecule follows that of other species. As well, matrix components including integrin alpha-6 (α6) and the hemidesmsome proteins, bullous pemphigoid antigen 1 (BP180) bullous pemphigoid antigen 2 (BP230) and plectin are also expressed.

**Conclusions:**

Isolation of equine keratinocytes and study of the matrix and adhesion related molecules produced by them provides a valuable tool for future work in the veterinary field.

## Background

The basement membrane (BM) is a thin layer of extracellular matrix (ECM) of which one of the major components is laminin (Ln). Laminins are a large family of heterotrimeric glycoproteins composed of at least 16 isoforms that play many roles in cell function, including cell adhesion and migration. Ln-332 (α3β3γ2) is a major isoform found in epithelial BMs [1].

Attachment of the epithelial cell to the underlying BM is mediated through hemidesmosomes (HD). The transmembrane integrin α6β4 links the epithelial cell to Ln-332 in the BM while the bullous pempigoid antigen 1 (BP180) also plays a role in cell attachment. The cytoplasmic proteins plectin and the bullous pemphigoid antigen 2 (BP230) connect the cytokeratin intermediate filament skeleton to the HD complex [2,3].

Extracellular matrix proteins are affected in diseases of multiple species. Recently, a mutation in the Ln-332 γ2 subunit in some Belgian horse foals has found to result in blistering of the skin, mouth epithelia and loss of the hooves [4]. A variety of human genetic and autoimmune bullous diseases also exist. Epidermolysis bullosa (EB) is a group of diseases resulting in blistering in the BM and skin fragility in which Ln-332, plectin, integrin α6, BP180 or collagen type VII may be affected [5]. As well, the bullous pemphigoid group of diseases characterized by subepidermal blistering and dysadhesion of epithelial cells, occur due to the presence of circulating antibodies against Ln-332 or BP180 [6,7]. Both lamellar BM and hemidesmosomal components are degraded during laminitis, a disease of the equine hoof with separation of the basal epithelial cell from the underlying BM along with degradation of the BM laminins and collagens [8-10].

Isolation and culture of keratinocytes from a variety of species has been described including human [11] and mice [12]. These methods have become well established through the development of specialized serum free media formulations, selective culture and substrate modifications which allow for successful routine cultivation of keratinocytes [13]. However, methods and specialized culture procedures for isolation and prolonged culture of equine keratinocytes, specifically from hoof lamellae, are less than optimal [14,15]. Such culturing of equine keratinocytes would provide a beneficial research tool for the *in vitro *study of laminitis and other epithelial related diseases in the horse. We modified tissue isolation and culture techniques to produce a method suitable for equine keratinocytes. Additionally, the *in vitro *production and processing of Ln-332 as well as production of other extracellular matrix proteins by equine keratinocytes were studied.

## Results

### Cell isolation and optimization of culture conditions

Skin keratinocytes isolated from lip epithelium were able to propagate on collagen type I coated substrate in DMEM supplemented with 5% FBS, 10 ng/ml EGF, 30 μg/ml BPE, 0.4 ug/ml hydrocortisone and 5 μg/ml insulin, at a calcium concentration of approximately 1.8 mM. Cells reached confluence in 7 ± 2.64 days (n = 3) and were sub cultured to passage 6 without significant loss of cell character.

**Figure 1:**
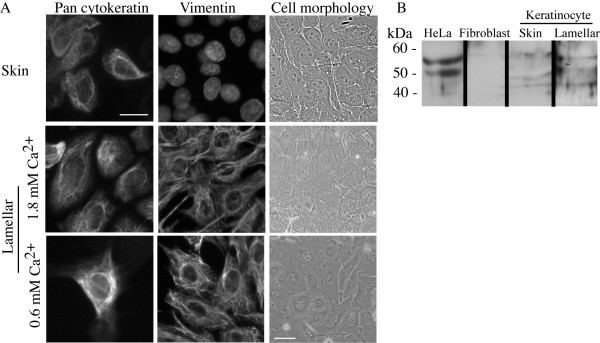
**Characterization of equine keratinocytes**. A. Immunofluorescence analysis of cytokeratin and vimentin expression in equine keratinocytes. Lamellar cells were grown in both high and low calcium conditions while skin keratinocytes are grown only in high calcium conditions. Nuclear staining with DAPI is shown for skin keratinocytes in middle panel, as these cells stained negative for vimentin Scale bar = 30 μm. Cell morphology images are phase contrast photographs of skin and lamellar cells. Scale bar = 10 μm. Images are representative of 3 independent experiments. B. Immunoblots of skin and lamellar whole cell lysates demonstrating expression of cytokeratins detected using antibody AE1/AE3 which recognizes both acidic and basic groups of cytokeratins. HeLa cell and equine skin fibroblast lysates are used as positive and negative controls, respectively, for cytokeratin expression. Images are representative of two independent experiments.

Initial studies found lamellar keratinocytes to reach 70 - 80% confluence under conditions optimal for skin keratinocytes, in a mean of 9.6 days ± 3.09 (n = 3), however cells were large, and unable to attach and proliferate upon sub-culture (Figure 1A, Table 1). A variety of other media conditions, in which calcium concentration was modified, were used to optimize growth for hoof lamellar cells. Calcium free DMEM media with supplementation with 5% FBS plus calcium (final concentration 0.6 mM) proved optimal from a variety of media compositions tested for lamellar propagation to passage 4 on collagen type I substrates (Table 1) resulting in homogenous populations of small cobblestone shaped proliferative cells (Figure 1A). Initial culture in lower calcium concentrations did not prove suitable for cell survival or proliferation upon sub culture. Additionally, equine keratinocytes were not able to grow or grew poorly in Keratinocyte Serum free media (KSFM) with serum supplementation, a media designed for isolation of human and mouse keratinocytes (Table 1).

**Table 1:** Optimization of culture conditions of lamellar keratinocytes

Growth Media	Calcium Concentration	Survival	Days to confluence (% confluence reached)	Ability to passage
KSFM + supp^a ^+2% FBS^b^	0.074 mM	No	14 (few cells remain attached)	N/D
KSFM + supp +5% FBS	0.275 mM	Yes	18 (20%)	N/D
DMEM -- Ca^2+^+ supp + 2% FBS	0.074 mM	No	16 (few cells remain attached)	N/D
DMEM -- Ca^2+^+ supp + 5% FBS	0.185 mM	Yes	19.5 ± 2.12 (70%) (n = 2)	No
DMEM -- Ca^2+^+ supp + 5% FBS	0.6 mM^c^	Yes	17.5 ± 0.71 (70%) (n = 2)	Yes

^a^10 ng/ml EGF + 30 μg/ml BPE + 0.4 μg/ml hydrocortisone + 5 μg/ml insulin

^b^FBS used had a calcium concentration of 3.7 mM (Thermo Scientific, Lot E08009)

^c^Media supplemented with additional calcium to 0.6 mM N/D = not determined

### Cell characterization

Equine keratinocytes demonstrated cobblestone cell morphology (Figure 1A) with few or no spindle shaped cells characteristic of fibroblasts. Expression of cytokeratins by both immunofluorescence and western blotting analysis was demonstrated (Figure 1A, B). HeLa cells served as a positive control, while equine fibroblasts served as a negative control (Figure 1B). The intermediate filament vimentin was expressed by only a few skin keratinocytes in these cultures while all lamellar cells expressed vimentin in the cell cytoplasm regardless of the calcium concentration of the growth medium (Figure 1A).

### Characterization of equine extracellular matrix components

**Figure 2:**
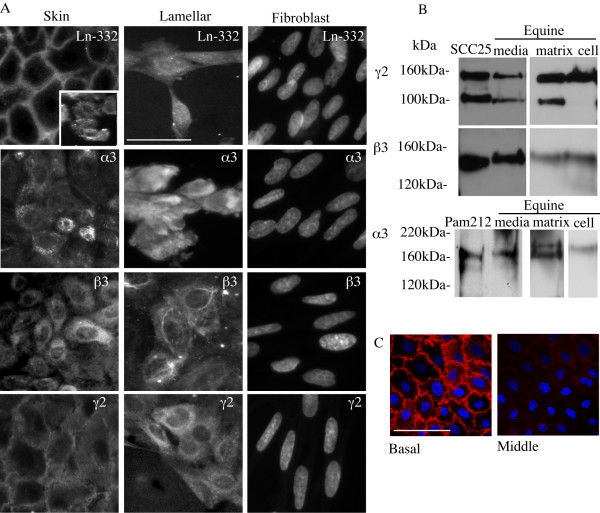
**Ln-332 synthesis and processing in equine keratinocytes**. A. Immunofluorescence analysis of expression of Ln-332 α3β3γ2 and individual Ln-332 subunits in skin and lamellar keratinocytes. Scale bar = 10 μm. Inset in skin keratinocytes panel A demonstrates Ln-332 α3β3γ2 localization in sub confluent cells compared to confluent cells in larger image. Images of equine fibroblasts stained with each antibody serves as a negative control. As these cells do not produce Ln-332, nuclear staining with DAPI is shown in each panel. B. Immunoblots of individual Ln-332 subunit forms present in cell and matrix layers as well as secreted media from equine keratinocytes. Aliquots of acetone precipitated conditioned media, cell layers and matrix preparations were analyzed with each Ln-332 subunit antibody. Serum free conditioned media of the human squamous cell line SCC25 and the mouse epidermal cell lime Pam212 serve as positive controls for Ln-332 subunit molecular weight and processing. C. Confocal microscopy of equine keratinocytes grown on coverslips labelled for Ln-332 α3β3γ2 localization. Images are shown from both the basal and middle section of the cell monolayer. Scale bar = 10 μm.

Ln-332 synthesized by equine keratinocytes was demonstrated by intracellular fluorescence of individual cells and small cell clusters (Figure 2A) while confluent monolayers show primarily intercellular staining (Figure 2A, inset). Likewise, individual Ln-332 subunits demonstrated intracellular staining in both skin and lamellar keratinocytes (Figure 2A). Equine fibroblasts did not show any significant expression of Ln-332 and thus confirm antibody specificity (Figure 2A). Immunoblotting analysis of individual cell and matrix layers and culture media demonstrated all three Ln-332 subunits are produced by equine keratinocytes intracellularly, and are deposited into the underlying matrix and secreted into the culture media (Figure 2B). The cell-associated form of the γ2 subunit corresponds to the full length 150-kDa subunit, while in both the deposited matrix layer and culture media an additional 105-kDa γ2' processed form was present. In all cell fractions, a 140-kDa band corresponding to the β3 subunit was present with no processed forms observed. The 190-kDa precursor α3 form and the processed 165-kDa α3 subunit forms were found in preparations of the deposited matrix. In conditioned media, the 165-kDa processed α3 subunit and a further processed form of 145-kDa was observed (Figure 2B). Confocal microscopy of cell monolayers demonstrated that Ln-332 is located primarily in the basal layer of confluent cells while little is observed on the apical or lateral layers (Figure 2C).

**Figure 3:**
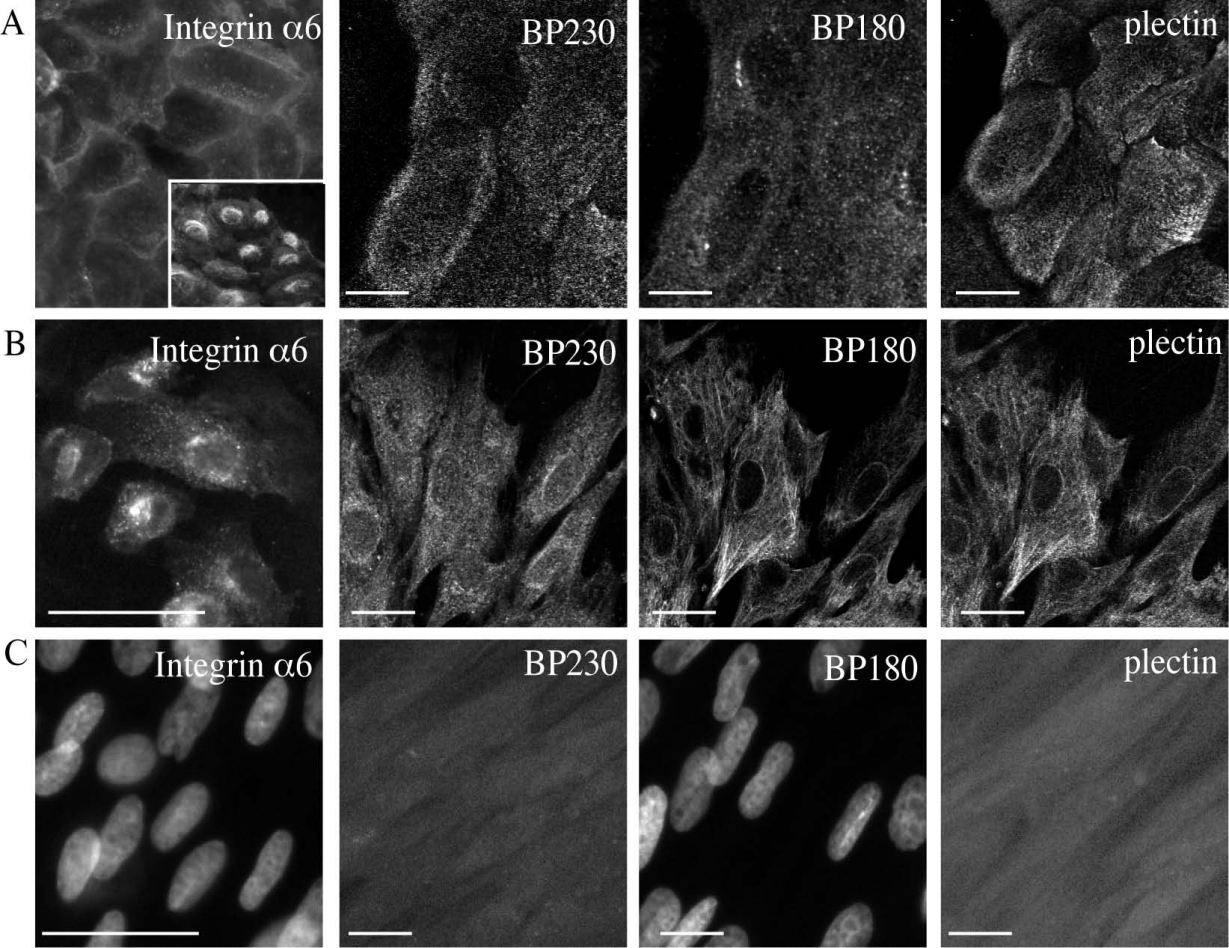
**Expression of hemidesmosome components in equine keratinocytes**. Immunofluorescence images of skin (A) and lamellar (B) keratinocytes and dermal fibroblasts (C) labelled for integrin α6 and proteins of the hemidesmosome complex. Cells were grown on coverslips, fixed and labelled with each antibody. Inset in skin keratinocytes panel A demonstrates integrin α6 localization in sub-confluent cells compared to confluent cells in larger image. Images are representative of three independent experiments. Scale bar = 20 μm.

By immunfluorescence microscopy, integrin α6 was localized in the cytoplasm of subconfluent cells (Figure 3A, inset) while in confluent cell monolayers staining was located intercellularly in a pattern identical to Ln-332 (Figure 3A, B). Hemidesmosome proteins, BP180, BP230 and plectin demonstrated a cytoplasmic punctate distribution in skin keratinocytes (Figure 3A), while in lamellar keratinocytes, a granular linear pattern was observed (Figure 3B). Equine fibroblasts demonstrated either no observable staining or faint diffuse background staining (Figure 3C).

## Discussion

Cultivation of primary cells is an essential technique for the *in vitro *study of a wide variety of cell types from many species. Similar to another report [16], skin keratinocytes isolated from lip epithelium were found to proliferate and propagate in culture. In the present study, cells were found to adhere well to collagen type I during the initial isolation and this substrate was used for initial growth in addition to subculture. Skin keratinocytes grew well in standard DMEM culture media with hormonal and serum supplementation and an approximate calcium concentration of 1.8 mM. This is in contrast to Dahm *et al *[16] who found media containing a calcium concentration of 0.6 mM optimal in their study, as with higher calcium they found early differentiation and loss of cell proliferation. Work presented here indicates that skin keratinocytes are able to proliferate at a higher calcium concentration, as long as suitable media and substrate conditions are present.

Initial studies to grow and passage hoof lamellar keratinocytes under conditions optimal for skin keratinocytes proved only minimally successful, possibly due to loss of cell proliferative capacity. Calcium concentration present in culture medium is known to play a major role in cell proliferation and differentiation [17]. For equine skin keratinocytes, a calcium concentration of 0.6 mM was found to be optimal for cell proliferation and propagation [16]. Likewise, for lamellar keratinocytes, growth in calcium free DMEM media supplemented with 5% FBS plus additional calcium (0.6 mM) proved optimal for prolonged lamellar cell propagation. These results show variation in the calcium requirements of keratinocytes from different tissues in the same species, as well as determining requirements for prolonged culture of primary equine lamellar keratinocytes.

As the epithelial and dermal lamellae are interlocked, easy separation of the two tissue types proves difficult and prevention of fibroblast overgrowth while maintaining keratinocyte proliferation proves difficult. Physical separation of the epithelial lamellae from the dermal lamellae using a modified hoof wall removal method resulted in little fibroblast contamination of cultures in our study, thus providing a simple method to isolate relatively pure populations of keratinocytes. The use of the protease dispase II during the isolation of skin keratinocytes, which separates epithelial tissue from the dermal tissue, also decreases fibroblast contamination [18]. An explant model of growth of bovine hoof keratinocytes at 37°C has been described, however without separation of the epidermal lamellae from the dermal tissue, cultures obtained were mixed and consisted predominately of fibroblasts with continued propagation [15]. Thus, the techniques used in our study provide simple methods to obtain relatively pure cultures of equine keratinocytes. The use of culture medium supplemented with a lower serum concentration of 5%, rather than standard 10%, was also used in an attempt to decrease fibroblast growth by minimizing serum derived factors which tend to stimulate fibroblast growth and inhibit epithelial cell proliferation [19]. Other culture medium additives including insulin [20], EGF [21], hydrocortisone [11] and bovine pituitary extract [22] have all been found to stimulate epithelial cell proliferation and appear to increase the growth of equine keratinocytes.

Similar to keratinocytes from other species, equine keratinocytes show characteristic cell morphology and express cytokeratins, the primary intermediate filament marker of the epithelial cell type. Along with filamentous cytoplasmic localization in cells by immunofluorescence, immunoreactive proteins between 46-kDa to 63-kDa were observed in cell lysates using an antibody recognizing acidic and basic cytokeratins similar to a study using equine lamellar tissue [23] as well as in HeLa cell lysates [24]. The intermediate filament vimentin was expressed by only a few skin cells in cultures and likely represented small numbers of contaminating fibroblasts confirmed by their elongated spindle shape morphology (data not shown). Vimentin has previously been thought of as a marker of cells of mesenchymal origin only, however human cultured keratinocytes have been demonstrated to express vimentin in low calcium conditions, yet when grown in higher calcium only cells located at the periphery of the cell outgrowth expressed vimentin [25]. We found all lamellar cells expressed vimentin in the cell cytoplasm regardless of the calcium concentration and did not display a fibroblastic cell morphology. Additionally, vimentin expression may be cell origin specific, as equine skin keratinocytes have been found to not express vimentin in culture [16] while lamellar keratinocytes in culture have been shown to express vimentin [14] in agreement with findings in this work.

Ln-332 plays a major role in attachment of the epithelial cell to the substratum through the interaction of the α3 subunit with integrin α6β4 as well as interaction between the β3 subunit with collagen and other laminin isoforms in the BM [26,27]. However, expression and processing of laminin isoforms in equine keratinocytes has not been studied extensively. Ln-332 expression *in vivo *has been demonstrated in the equine hoof [9] and skin [4,9] localized to the BM. This study has confirmed that equine skin and hoof keratinocytes cells produce Ln-332 *in vitro*.

Ln-332 is synthesized intracellularly to form a α3β3γ2 heterotrimer and following secretion from the cell and incorporation into the ECM, the human γ2 subunit is converted from 150-kDa to 105-kDa while the α3 subunit is converted from 190-kDa to 165-kDa [28]. This study confirms that equine Ln-332 subunits are synthesized and processed in a similar manner. Overall, skin and lamellar keratinocytes showed similar Ln-332 expression and processing confirming that the same events occur regardless of the tissue of origin.

Integrin α6 showed the same expression pattern similar to Ln-332 localization in equine cells. This would be expected as Ln-332 binds to integrin α6 to provide cell attachment and corresponds to what is seen in other cell types [29,30]. Expression of the hemidesmosome proteins BP180, BP230 and plectin in skin keratinocytes showed a cytoplasmic punctate distribution similar to other species [31-34]. Alternatively, granular linear staining patterns of localization have also been observed for these proteins in some cell lines [35,36] including lamellar keratinocytes. The reasons for this difference between cell origin are unclear, however as BP230 and plectin function to interact with the cytoskeleton, localization similar to intermediate filaments is not unexpected.

## Conclusions

In this study we have refined methods for prolonged culture and subculture of equine keratinocytes and have provided evidence that both skin and hoof lamellar keratinocytes produce Ln-332 and hemidesmosome proteins *in vitro*. Expression and processing patterns are similar to their counterparts from other species and not only provides a basis for further study but also extends knowledge in the field of matrix research. The methods developed in this study will allow for easier analysis and more manageable models of cell types and factors involved in equine diseases as well as provide a starting point for the development of a physiological *in vitro *model system of the unique equine lamellar structure.

## Methods

### Tissue collection

Hoof and skin tissue was collected from clinically normal horses euthanized for unrelated reasons. Experiments were conducted according to the animal ethics guidelines set by The University of Queensland Animal Ethics Committee.

### Isolation of equine keratinocytes

Keratinocytes were isolated from lip epithelium as described [14] with the following modifications. Tissue pieces were incubated in 2.4 U/ml Dispase II (Roche) 18 h at 4°C followed by separation of the epidermis from the dermis. The epithelial tissue was minced with a scalpel and incubated in 0.25% trypsin with 0.05% EDTA (Invitrogen) 30 min at 37°C, 100 rpm, three times, followed by pooling of cells and trypsin inactivation by the addition of serum containing medium. Cells were washed and grown on type I collagen (Becton Dickinson) in DMEM supplemented with 5% FBS, 30 μg/ml bovine pituitary extract (BPE), 10 ng/ml EGF, 5 μg/ml insulin (Invitrogen) and 0.4 μg/ml hydrocortisone (Sigma) at a density of 1.5 × 10^5 ^cells/cm^2 ^for 48 h followed by media replacement. Cells were sub-cultured using an initial wash with 0.02% EDTA to remove any contaminating fibroblasts, removed with 0.25% trypsin and reseeded at a density of 5 × 10^4 ^cells/cm^2^.

For isolation of hoof lamellar keratinocytes, the distal forelimb was disarticulated at the carpal joint and the hoof was soaked in 10% sodium hypochlorite, followed by 70% ethanol. Hoof wall strips were removed by cutting through the distal hoof wall between the coronet and toe as described [37]. Epithelial tissue remaining attached to the separated hoof wall was removed and cells isolated as described for skin keratinocytes. Cells were grown in either keratinocyte serum free media (KSFM) or calcium free DMEM (Invitrogen) with 10 ng/ml EGF + 30 μg/ml BPE + 0.4 μg/ml hydrocortisone + 5 μg/ml insulin. Additionally, media was supplemented with 2% or 5% FBS alone (Thermo Scientific, lot E08009) or with additional calcium to 0.6 mM. Cells were propagated and sub-cultured as described above.

### Isolation of equine fibroblasts

Fibroblasts were isolated from dermal tissue remaining from skin keratinocyte isolation. Dermal tissue was finely minced and trypsinised as for skin keratinocytes above. Cells were collected and grown on tissue culture plastic in DMEM supplemented with 10% FBS at a density of 2 × 10^4 ^cells/cm^2^.

### Conditioned media and cell lysate preparation

Conditioned media from the human squamous cell carcinoma cell line SCC25, [38] (a gift from Dr. Nicholas Saunders, University of Queensland) was prepared as described [39,40], while conditioned media from the mouse epidermal cell line Pam212 was a gift from Dr. Takako Sasaki (Shriners Hospital for Children Research Center). Conditioned media samples from these cell lines were used as positive controls for Ln-332 subunit molecular weights and processing events on immunoblots.

Equine keratinocyte conditioned media was prepared from cells grown 48 h in serum free medium followed by concentration by acetone precipitation (1 volume media: 6 volumes acetone) or centrifugation filter devices (Centricon, 10,000 MWCO, Millipore).

Lysates of HeLa cells to serve as positive controls for cytokeratin immunoblots were a gift of Dr. Jennifer Stow (University of Queensland).

### Antibodies

The Ln-332 γ2 domain III subunit polyclonal antibody, Pab26, was a gift from Dr. Karl Tryggvason (Division of Matrix Biology, Karolinska Institute). Antibodies K140 and H300 directed toward the Ln-332 β3 subunit were kind gifts of Dr. Peter Marinkovich (Program in Epithelial Biology, Stanford University) and Dr. Guerrino Meneguzzi (INSERM, Faculte de Medecine) respectively. Ln-332 α3 subunit antibodies BM165, pSE585 and 1118 were kind gifts of Dr. Peter Marinkovich and Dr. Takao Sasaki respectively. A rabbit polyclonal antibody, ab14509 (Abcam), directed to all three Ln-332 subunits was also used.

The monoclonal antibodies 5E recognizing BP230 and 417D1 recognizing plectin as well as the polyclonal antibody J17 directed against BP180 were kind gifts of Dr. Jonathan Jones (Department of Cell and Molecular Biology, Northwestern University). GOH3, a monoclonal antibody directed to integrin α6, was a gift from Dr. Arnoud Sonnenberg (The Netherlands Cancer Institute).

The pan-cytokeratin antibodies, MNF116 (cytokeratin 5, 6, 8, 17, 19, DAKO) and AE1/AE3 (acidic and basic groups, Zymed) and the vimentin antibody V9 (Zymed) were also used.

### Immunofluorescence Labelling

Keratinocytes were grown on glass cover slips coated with collagen type I (5 μg/cm^2^, Sigma) or cytocentrifuged onto poly-L-lysine slides (Shandon 4 cytospin, Thermo Shandon) while fibroblasts were grown directly on glass coverslips, followed by fixation in 4% paraformaldehyde for 90 minutes at room temperature and permeabilisation with 0.1% Triton X-100 for 5 minutes or fixed in -20°C methanol for 5 minutes. Cells were incubated with primary antibodies diluted in 5% normal goat serum followed by incubation with fluorescent-conjugated secondary antibodies (Molecular Probes, Jackson Laboratories). Samples were viewed using either epifluorescence (Olympus BX-50 microscope equipped with a BX-FLA reflected light fluorescence attachment (Olympus Optical Company)) or confocal fluorescence microscopy (Zeiss LSM 510 META microscope (Carl Zeiss Microscope Systems)).

### SDS-PAGE and Immunoblotting

Total cell lysates [16] and individual cell and matrix layers were prepared [29]. Samples were separated using 8-16% gradient SDS- PAGE mini gels (NuSep) followed by transfer to poly-vinyl-D-fluoride membrane (GE healthcare). Membranes were blocked in 5% skim milk powder/0.1% Tween-20 in PBS followed by incubation with primary antibody and subsequent horseradish peroxidase-conjugated secondary antibody (Zymed) with chemiluminescent detection (Super Signal, Pierce).

## Authors' contributions

MBV designed the study, carried out all experiments and drafted the manuscript. CCP participated in design of the study and helped draft the manuscript. All authors read and approved the final manuscript.
